# The NBS Scale Of Radiance Temperature

**DOI:** 10.6028/jres.092.002

**Published:** 1987-02-01

**Authors:** William R. Waters, James H. Walker, Albert T. Hattenburg

**Affiliations:** National Bureau of Standards, Gaithersburg, MD 20899

**Keywords:** calibrations, gold-point blackbody, radiance temperature, response linearity, standards, tungsten-strip lamps

## Abstract

This paper describes the measurement methods and instrumentation used in the realization and transfer of the International Practical Temperature Scale (IPTS-68) above the temperature of freezing gold. The determination of the ratios of spectral radiance of tungsten-strip lamps to a gold-point blackbody at a wavelength of 654.6 nm is detailed. The response linearity, spectral responsivity, scattering error, and polarization properties of the instrumentation are described. The analysis of sources of error and estimates of uncertainty are presented. The assigned uncertainties (three standard deviations) in radiance temperature range from ±2 K at 2573 K to ±0.5 K at 1073 K.

## 1. Introduction

Temperatures above the freezing point of gold (1337.58 K) are defined on the International Practical Temperature Scale (IPTS-68) [1]^1^ in terms of the ratio of spectral radiances of two blackbody sources, one of which is maintained at the gold point. Thus, the accurate measurement of the spectral radiance ratio of two sources is required for the realization and dissemination of the radiance temperature scale. Currently, such measurements are performed at NBS using a spectroradiometer operated by a computer-controlled data acquisition system, which permits rapid comparison of the spectral radiances from a variety of sources. With this system, lamp standards of radiance temperature are calibrated in the range 1337.58 K to 2573 K. For convenience, the calibrations are extended to 1073 K, where the scale is defined in terms of thermocouples. Calibration services are also available for optical pyrometers. The purpose of this paper is to provide a detailed description of the instrumentation and procedures used to realize and disseminate the radiance temperature scale. Detailed information on the calibration services appears in a separate report [2].

## 2. Basic Theory

Spectral radiance is the radiant power contained in a defined area, solid angle, and wavelength interval,

$$L_{\lambda }=d^{3}\Phi /dA\cdot \cos\Theta \cdot d\Omega \cdot d\lambda$$ (1)

where *L*_λ_ is the spectral radiance, Φ is the radiant flux, *A* is the area, Θ is the angle between the surface normal and the direction of propagation, Ω is the solid angle about that direction, and λ is the wavelength. The relation between spectral radiance, wavelength and temperature is given by Planck’s Law,

$$L_{\lambda }=c_{1}/\pi \cdot \lambda^{5}\cdot [\exp(c_{2}/\lambda \cdot T)-1]$$ (2)

where *c*_1_ and *c*_2_ are the first and second radiation constants, λ is the wavelength in vacuo, and *T* is the temperature. The defining equation for the IPTS above 1337.58 K is therefore

$$\begin{array}{l} r=L_{\lambda }(T)/L_{\lambda }(T_{\text{Au}})\\ \;=[\exp(c_{2}/\lambda \cdot T_{\text{Au}})-1]/[\exp(c_{2}/\lambda \cdot T)-1] \end{array}$$ (3)

where *L*_λ_(*T*) and *L*_λ_(*T*_Au_) are the spectral radiances of the two blackbodies at temperatures *T* and *T*_Au_, *T*_Au_ is the temperature of freezing gold defined as 1337.58 K, r is their ratio, and *c*_2_ is defined as 1.4388 cm-K. In principle, a measurement of the ratio at a discrete wavelength with a linear response instrument yields the value of *T*.

In practice, the radiance temperature scale is realized with an instrument of finite bandpass, and an integral form of eq (2) must be used.

$$r=\int L_{\lambda }(T)\cdot R_{\lambda }\cdot d\lambda /\int L_{\lambda }(T_{\text{Au}})\cdot R_{\lambda }\cdot d\lambda$$ (4)

where *R*_λ_ is the spectral responsivity of the instrument and includes the spectral transmittance of the wavelength-limiting element (e.g., interference filter or monochromator), the spectral transmittance of all other optical elements, and the spectral responsivity of the detector. A determination of *R*_λ_ allows a calculation of *T* from eqs (2 and 4). Determination of the ratio for a range of values provides a temperature scale over the corresponding temperature range.

The radiance temperature scale is typically maintained and disseminated on tungsten-strip lamps, which possess a repeatable current vs. radiance- temperature relationship not available in present variable-temperature blackbodies. Realization of the scale with these lamps and a gold-point blackbody is also practical, and is the typical procedure followed by standards laboratories [3]. This scale is valid only for the wavelength of realization, since the spectral distributions of the lamps are not known functions. Traditionally the wavelength of realization has been near 650 nm, a convenient region for visual optical pyrometers. At NBS, a spectroradiometer system is presently being used and a wavelength of 654.6 nm (wavelength of a thorium spectral line) has been chosen. This method requires that the instrument relative spectral responsivity function extend only over an acceptably small spectral range, or is known accurately enough to determine the wavelength at which the integrands of eq (4) have the same ratio as the integrals.

## 3. Measurement Apparatus

**Figure 1:**
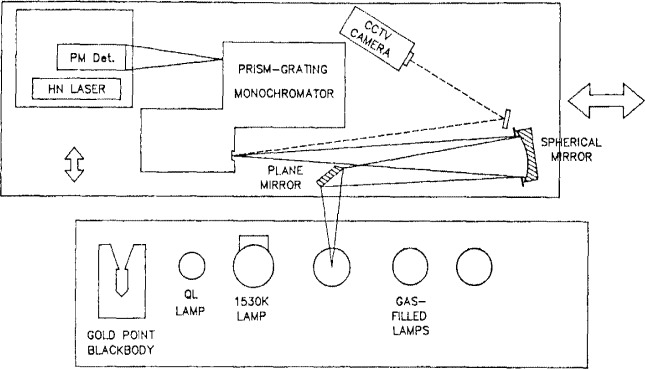
FASCAL radiance temperature measurement.

**Figure 2:**
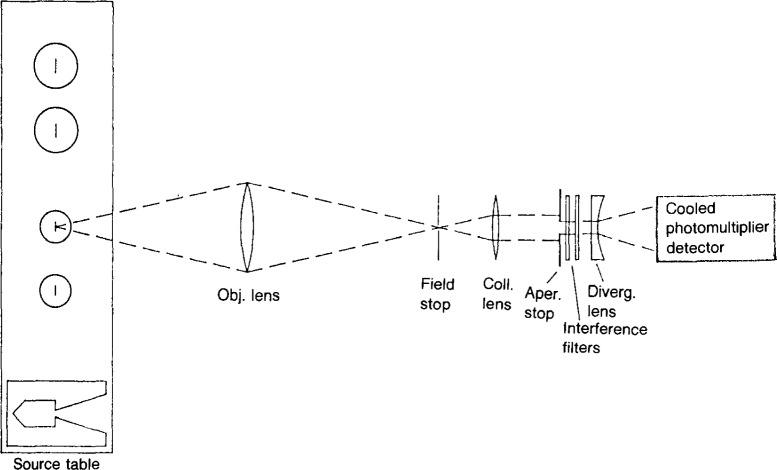
Schematic of photoelectrie pyrometer.

The radiance temperature calibrations described in this report are performed on the NBS Facility for Automated Spectroradiometric Calibrations (FASCAL). This system employs a prism-grating double monochromator, mirror optics, a highly stable external tungsten reference source, and a photomultiplier tube operated in a linear response mode. A block diagram of the system is shown in figure 1. Prior calibrations were carried out on the NBS Photoelectric Pyrometer [4]. A new version of this instrument is under development, which will employ interference filters (1 nm bandpass) and refractive optics. The measurement process will be nearly identical to that of the FASCAL system, utilizing an external reference source and a linear- response photomultiplier tube. A schematic diagram of the new instrument is shown in figure 2.

### 3.1 Gold-Point Blackbody

The Gold-Point Blackbody (GPBB) is a graphite cylinder with a small viewing hole (diameter 1 mm) at one end, and a conical cavity at the other. The cylinder is surrounded by 0.99999 pure gold, and the crucible containing the gold is surrounded by heating coils and an insulated case. The construction and characterization of the GPBB have been detailed in prior reports [4]. The estimated emissivity is 0.9999. The duration of a melt or freeze plateau is approximately 5 minutes, and the time delay between these observation periods is about 5 minutes.

### 3.2 Lamp Sources

Tungsten-strip filament lamps are used both in the realization of the radiance temperature scale and as secondary standards for scale dissemination. Each filament has a small notch in one edge, about midway along its length, to aid in determination of the filament portion to be calibrated (target area). A small mark is placed on the rear of the lamp envelope to permit reproducible angular positioning.

#### 3.2.1 Vacuum Lamps

Vacuum tungsten-strip lamps of the Quinn-Lee type [5,6] are used in the scale realization. One lamp is operated at a single current to produce a spectral radiance equal to that of the gold-point blackbody at 654.6 nm. A second lamp is operated at a single current to produce a spectral radiance of about eight times that of the gold-point blackbody at 654.6 nm (about 1530 K radiance temperature). Both lamps are stable to better than 0.02% over 200 burning hours when operated under these single-level conditions.

#### 3.2.2 Gas-Filled Lamps

Gas-filled tungsten-strip lamps are calibrated as secondary standards for dissemination of the radiance temperature scale. Prior to calibration, the lamps are annealed at a radiance temperature of 2350 °C for 2 hours on direct current. Each lamp is examined for the variations in spectral radiance with angle of emission, while set at a radiance temperature of 1700 °C. The lamp orientation is chosen to minimize variations in spectral radiance with rotation, and an alignment arrow is then etched on the rear of the envelope. The amount and orientation of polarization effects are also determined. Lamps selected for calibration display a degree of polarization of 0.005 or less.

### 3.3 Spectroradiometer

#### 3.3.1 Fore-optics

Sources are imaged with unit magnification at the entrance slit of the monochromator by the two front-surface aluminized mirrors shown in figure 1. The mirror surfaces are stripped and recoated at intervals to reduce signal loss due to oxidation. The plane mirror directs the beam to the spherical mirror (radius of curvature 1220 mm) along a line about 1.5 degrees from the spherical mirror axis. The spherical mirror focuses the beam onto a mirror-surfaced mask, placed immediately in front of the entrance slit. The mask is engraved with horizontal and vertical scales with 0.1 mm divisions, and is viewed at high magnification by either a telescope or a video camera to allow for precise positioning of the sources. The mask determines the height of the system field stop (source target area). The entrance slit determines the width. The stop dimensions are 0.8 mm high by 0.6 mm wide for the scale realization and transfer. The system aperture stop is located within the monochromator.

#### 3.3.2 Monochromator

A prism-grating double monochromator is employed to minimize spectral scattering and avoid multiple orders. The dispersion is about 4 nm/mm at the 654.6 nm wavelength setting. The entrance aperture (solid angle) is rectangular in shape, with a vertical angle of 0.125 radians and a horizontal angle of 0.0625 radians. The wavelength setting is calibrated against a spectral line standard (thorium) and is repeatable to within 0.05 nm. The entrance, intermediate, and exit slits are adjustable from 0.01 to 3.0 mm as a unit, resulting in a nearly triangular-shaped spectral responsivity function.

#### 3.3.3 Detector

For the radiance temperature determinations an end-on 11-stage photomultiplier with quartz window and S-20 spectral response is placed behind the exit slit. The detector is cooled to 258 K with a thermoelectric cooler. The anode current is amplified and converted to a 0–10 volt signal by a programmable amplifier.

### 3.4 Control and Data Acquisition System

The FASCAL system employed for the radiance temperature calibrations permits control of the entire measurement process from a remote operator console after initial source alignment. Component positions, instrument settings, sequence of operations, and data collection are effected by either stored computer programs, operator commands, or a combination of the two.

The system is directed by a microcomputer and a high-speed disk system for program and data storage. A modular interface controller [7] provides the link between instruments and computer. All measurement signals are multiplexed into the digital voltmeter through the interface scanner, and the instruments are remotely programmed and controlled through interface modules. All instrument settings and signal outputs are printed and stored on disk for later analysis. The spectroradiometer (fore-optics, monochromator, and detector) and a closed-circuit TV camera are mounted on a carriage. The carriage can be positioned by remote command along a linear track, to align the spectroradiometer with one of the sources mounted at fixed stations along the track. The average move time between stations is a few seconds, and positions are repeatable to about 0.1 mm. The TV camera presents a highly magnified image of the monochromator entrance slit mask to video displays at the spectroradiometer and at the operator console for initial source alignment and subsequent monitoring.

## 4. Measurement of Instrument and Source Parameters

### 4.1 Spectral Responsivity Function

The relative spectral responsivity function of the spectroradiometer is determined by the indirect method [8]. In this method, the relative responsivity function is treated as the product of two terms, the responsivity factor and the slit-scattering function, where the responsivity factor depends only upon the wavelength of the observed flux and the slit-scattering function depends only upon the difference between the wavelength setting of the monochromator and the wavelength of the flux. This factorization of the spectral responsivity function is valid if the instrument dispersion, aberrations, scattering, and diffraction are constant over the wavelength region of interest. This assumption is valid in the central portion of the relative responsivity function, but values for the distant wings are subject to error due primarily to changes in scattering and dispersion.

The responsivity factor is obtained by spectrally scanning a continuous source standard of spectral radiance with narrow (0.1 mm) slits. To determine the slit-scattering function, an integrating sphere irradiated by a krypton or argon laser is spectrally scanned by the spectroradiometer, with the slit widths set at the 0.6 mm width used in the scale realization and transfer. The plot of the output signal versus wavelength is the mirror image of the plot of the slit-scattering function versus wavelength [8]. For a 647 nm krypton laser, the function is nearly triangular in shape with a width at half- height of 2.2 nm. Relative to the peak value, the measured values decrease to about 10^−3^ at 3 nm, 10^−4^ at 15 nm, and 10^−7^ at 70 nm from the central wavelength. At 150 nm from the central wavelength, the value decreases to 10^−8^ in the short- wavelength wing and to 10^−9^ in the long- wavelength wing. Scans with 488 and 514 nm (argon), and 676 nm (krypton) yield similar results. These values were confirmed over the central and near wings portion of the function by measurements with the direct method, using a dye laser tuned through a series of wavelengths with the spectroradiometer set at a fixed wavelength [9]. Since the function changes very slowly with wavelength in the visible region, the measurement at 647 nm yields the slit-scattering function at 654.6 nm.

### 4.2 Linearity of Response

The degree of linearity of the spectroradiometer response is determined with an automated beam conjoiner [10]. A beam from a constant source is split into two branches, whose fluxes are independently attenuated or blocked before recombination and further attenuation. The flux contribution from both branches is equal to the sum of the fluxes from each branch when measured separately (additivity). The device provides 96 levels of flux over a range of about 1 to 500. The levels are presented in random order to avoid systematic errors, and are interspersed with 29 zero flux levels. A microcomputer controls the attenuating filters and records the filter positions and radiometer signals. The data is least-squares fitted to a polynomial response function, to determine a correction factor by which the radiometer output signal must be multiplied to obtain a quantity proportional to radiant flux.

The measured instrument response is linear to within ±0.2% for a range of photomultiplier anode currents from 1 to 500 nanoamperes. For currents much less than 1 nanoampere, the signal is limited by noise. For currents greater than 1 microampere, the linearity correction increases rapidly, rising to 3% at 7 microamperes. The anode current is restricted to less than 500 nanoamperes during measurements by selection of appropriate photomultiplier tube voltage. Correction factors for the amplifier ranges are determined from measurement of a known electrical current and combined with the linearity correction factor.

### 4.3 Polarization

The polarization properties of the spectroradiometer and the gas-filled lamps are measured with the aid of dichroic (linear) polarizers positioned in motorized rotating mounts. The sheet polarizer properties and those of the spectroradiometer are determined in an initial set of experiments, using an illuminated integrating sphere as a source of unpolarized radiation. The characterized polarizer and spectroradiometer are then used to measure the polarization properties of the lamp sources.

The determination of spectroradiometer and sheet polarizer properties consists of spectral radiance measurements of the sphere source alone, with a pair of similar polarizers interposed in the beam and set at a number of angular positions (rotations about the optic axis), and with each polarizer individually placed in the beam at the same angular positions. In order to account for departure from ideal behavior, a Mueller transmittance matrix [11] containing six parameters is assumed for the sheet polarizers. Circular polarization is assumed to be negligible. The spectroradiometer polarization direction is determined in a preliminary experiment and chosen as the polarization reference direction, leaving only the degree of polarization to be determined for the instrument. Our measurements provide about 200 equations involving 10 unknowns, whose values are then obtained by a non-linear least-squares solution.

The sphere source is replaced by the lamp source whose properties are to be measured using a characterized sheet polarizer and spectroradiometer. Measurements are made with the lamp alone and with the polarizer set at the same angular positions as before. This results in 25 equations involving the two source unknowns, whose values are obtained by a least-squares solution. The polarization of the lamp source is specified by the direction of maximum linear polarization *τ*, and the degree of polarization

$$P=(L_{\max}-L_{\min})/(L_{\max}+L_{\min})$$ (5)

where *L*_max_ and *L*_min_ are the maximum and minimum readings of a polarization-indifferent radiometer when an ideal linear polarizer is rotated in the source beam. The factor (see Appendix) required to reduce the signal ratio of eq (4) to a value which would be obtained with a polarization-indifferent radiometer is

$${\left(1+Bp_{1}\right)}^{-1}$$

where *p*_1_=p·cos2*τ* and *B* is the spectroradiometer polarizance (degree of linear polarization introduced by the spectroradiometer). For the spectroradiometer employed here, the measured polarizance is 0.26 at 654.6 nm. The degree of polarization of a typical lamp selected for calibration is about 0.003. The uncertainty in the radiometer polarizance *B* is estimated as ±0.0005, and the lamp polarization uncertainties are estimated as ±0.002 (uncertainties are stated at the three standard deviations level).

### 4.4 Size of Source

The “size of source” effect (signal contribution due to flux which originates outside the target area and is scattered into the measured beam by the fore-optics) is determined by observing the signal from a 0.6 by 0.8 mm segment of a uniform diffuse source, and noting the change in signal when the surrounding area of the source is changed by placing various masks on the diffuse source. The masks expose source areas which closely approximate the radiant areas of the lamp and blackbody sources used in the scale realization and transfer. As a check, the effect is also evaluated by observing changes in the near-zero signal from a “black hole” (an absorbing cavity slightly larger than the 0.6 by 0.8 mm field stop) as the various surrounding area masks are positioned. The observed differences are used to apply a correction to the signals observed in source comparisons. The measured effect varies from 0.04% to 0.1% at 654.6 nm depending upon the elapsed time since the last mirror recoating.

## 5. Process of Realization and Transfer

The process of realization and transfer consists of three steps, all carried out at a wavelength of 654.6 nm. In step one, the gold-point spectral radiance is transferred to a Quinn-Lee vacuum lamp. In step two, a second vacuum lamp at about 1530 K radiance temperature (about eight times the gold- point spectral radiance) is compared to the first vacuum lamp. In step three, the second lamp is compared to the test lamp. After resetting the test lamp current, step three is repeated for each desired radiance temperature. If the vacuum lamps remain constant between observations, the product of the signal ratios from the three comparisons is the signal ratio of the test lamp to the GPBB. With appropriate corrections for size of source and polarization, the spectral radiance and thus the radiance temperature of the test lamp can be evaluated from eqs (4 and 2). The polarization and the spectral radiance distributions over Ω, *A*, and λ of the vacuum lamps are of no concern here, since the lamps serve only to reproduce the spectroradiometer signal between comparisons. The only requirement is that the vacuum lamp parameters remain constant between comparisons with the gold-point blackbody (GPBB) and with the test lamp. This is satisfied by the constant-current operation of the stable (0.02% per 200 hours) vacuum lamps. Comparison with the GPBB is performed about every 50 to 100 hours of lamp operation, and the vacuum lamps are compared with each other weekly during experiments. The use of the first vacuum lamp avoids the inconvenience of manipulating the GPBB through its melting and freezing point cycles and provides a continuous measure of goldpoint radiance. It also increases the number of experiments allowed between maintenance or replacement of the gold-point furnace. The use of the second vacuum lamp keeps all signal ratios within the linear response region of the spectroradiometer (i.e., within the 1 to 500 nanoampere range of anode current).

## 6. Data Analysis and Uncertainties

### 6.1 Temperature Values

The signal ratio of the test lamp to the GPBB is obtained from the product of the signal ratios measured in the first vacuum lamp comparison with the GPBB, the second (1530 K) vacuum lamp comparison to the first, and the test lamp comparisons to the second lamp. The ratio is then multiplied by correction factors to account for the size-of-source disparity, polarization error, and departure from linear response where appropriate. An estimated test lamp radiance temperature is then calculated from the ratio of Planck functions for two black- bodies which have the same signal ratio (one blackbody at the gold point), using 654.6 nm as the wavelength. This temperature can then be used to determine by iteration the exact temperature which will satisfy eq (4). For the spectral responsivity function of this spectroradiometer, this exact temperature differs from the estimated temperature obtained from the Planck ratio by an amount which is small (less than 0.2 K) and is a simple function of the temperature. Therefore, to avoid the repetitive iteration process, the temperature calculated from the Planck ratio is corrected to the desired eq (4) value by this known difference.

### 6.2 Uncertainties

The uncertainties in the radiance temperature values assigned to the calibrated lamps are obtained from the observed imprecision of the measurements and the estimated systematic error in both the measured and the provided quantities (e.g., temperature of melting gold). Uncertainties obtained from observed imprecision and from published values for the physical constants are based upon three standard deviations. Uncertainties of systematic errors are estimated at the equivalent of three standard deviations.

In order to examine the contributions of the various errors to the uncertainty in radiance temperature, an approximate equation for the complete measurement process can be developed by using the Wien approximation to eq (2),

$$L_{\lambda }≅(c_{1}/\pi )\cdot \lambda^{-5}\cdot \exp(-c_{2}/\lambda \cdot T)$$ (6)

to express the spectral radiance of a blackbody. With this approximation, eq (3) becomes

$$\begin{array}{l} r=\exp(c_{2}/\lambda \cdot T_{\text{Au}})/\exp(c_{2}/\lambda \cdot T)\\ \;=\exp[(c_{2}/\lambda )\cdot (1/T_{\text{Au}}-1/T)]. \end{array}$$ (7)

Solving for *T*, and expressing r in terms of the measured ratios and their correction factors, we can express the complete measurement process as

$$T={\left\{(1/T_{\text{Au}})-(\lambda /c_{2})\cdot \ln[s\cdot d\cdot f\cdot M_{0}\cdot M_{1}\cdot M_{2}/(1+B\cdot p_{1})]\right\}}^{-1}$$ (8)

where the definitions of the quantities and their estimated 3*σ* uncertainties are:

*M*_0_, signal ratio GPBB vs. first vacuum lamp (0.12%)
*M*_1_, signal ratio first vacuum lamp vs. 1530 K lamp (0.12%)
*M*_2_, signal ratio 1530 K lamp to test lamp (0.2–0.5%)
*s*, size-of source correction for GPBB vs. test lamp (0.1%)
*d*, correction for test lamp drift during calibration (0.1%)
*f*, linearity-range factor correction (0.04–0.1%)
*T*_Au_, IPTS-68 temperature of freezing gold (0.4 K)
*c*_2_, second radiation constant (0.00014 cm·K)
*B*, spectroradiometer polarizance (0.05%)
*p*_1_, lamp polarization component (0.2%)
λ, wavelength setting at 654.6 nm (0.15 nm)

Radiance temperature uncertainties due to the factors of eq (8) are obtained from the partial derivative with respect to those factors and the estimated uncertainty in the factor (propagation of error). For example, the calculated uncertainty in radiance temperature at the 2300 °C (2573.15 K) point due to the 0.4 K uncertainty in *T*_Au_ is

$$\Delta T={\left(2573.15/1337.58\right)}^{2}\cdot (0.4)=1.48\;K.$$

Differences between errors calculated by eq (8) and those calculated by the exact Planck equation are negligible. Note that for the wavelength λ this process yields the error due to inserting the wrong wavelength in the calculation, not the error due to incorrect wavelength setting.

In addition to the factors which appear explicitly in this relation, uncertainties in the ratios *M*_0_, *M*_1_, and *M*_2_ arise from errors in the wavelength setting λ, in the current measurements of the vacuum (0.2 ma) and gas-filled (2 m*a*) lamps, in the alignment of lamps and in the measured spectral responsivity function. The uncertainties in the ratios due to wavelength setting and current are assessed by measurement of the change in signal ratio when varying these quantities. The effect upon the signal ratios due to the uncertainty in the measured spectral responsivity function is determined by solving eq (4) for a range of *R* (λ_0_, λ) values, using the known spectral radiance distribution of the GPBB and an approximate test lamp distribution derived from spectral scans of such lamps. The radiance temperature uncertainties due to these factors are then deduced from the ratio uncertainties as before. The uncertainties in signal ratio, wavelength setting, lamp currents and lamp alignment are considered random errors; the remaining errors are systematics. Table 1 summarizes the uncertainties obtained by this process.

## Figures and Tables

**Table 1:** Summary of estimated uncertainties in degrees C.

Source of Uncertainty	Temperature (°C)				
	800	1100	1400	1800	2300
Signal Ratio *M*_0_ (r)	0.06	0.10	0.15	0.23	0.36
Signal Ratio *M*_1_ (r)	0.06	0.10	0.15	0.23	0.36
Signal Ratio *M*_2_ (r)	0.27	0.27	0.28	0.37	0.67
Size of Source (s)	0.05	0.09	0.13	0.20	0.30
Lamp Drift (s)	0.05	0.09	0.13	0.20	0.30
Linearity (s)	0.05	0.03	0.05	0.13	0.30
Temperature Freezing Gold (s)	0.26	0.42	0.62	0.96	1.48
Second Radiation Constant (s)	0.02	0.0	0.04	0.11	0.22
Polarization (s)	0.04	0.06	0.09	0.14	0.21
Wavelength Setting (r)	0.08	0.05	0.08	0.20	0.44
Vacuum Lamp Current (r)	0.05	0.08	0.13	0.18	0.29
Test Lamp Current (r)	0.29	0.18	0.12	0.09	0.07
Spectral Responsivity (s)	0.03	0.01	0.0	0.02	0.03
Lamp Alignment (r)	0.13	0.21	0.32	0.49	0.76

Total estimated 3*σ* uncertainty on Thermodynamic Scale (square root of sum of squares)	0.5	0.6	0.8	1.3	2.0

Note: Random errors denoted by (r), systematic by (s).

## Footnotes

1 Figures in brackets indicate literature references.

## APPENDIX

### Measurement of Instrument and Source Polarization

The measurement of source polarization and the determination of its effect on the measurement of spectral radiance is carried out with a sheet polarizer mounted in a motorized rotating mount. This mount fits a slot milled into a bracket attached to the front of the spectroradiometer. By this means the polarizer can be reproducibly positioned in the same location between the source and the spectroradiometer whenever required.

Before the polarizer can be used for source polarization measurements its polarization and that of the spectroradiometer must be measured. This is accomplished with the help of an integrating sphere and a second polarizer similar to the first. The integrating sphere is mounted in the radiometer source plane so that its exit port serves as a source of unpolarized radiance. Its entrance port, located at about 90° from the exit port, is irradiated with a suitable light source, such as a quartz-halogen lamp. Then a series of spectral radiance measurements of this source is recorded: a) with no polarizer, b) with the first polarizer in its slot, c) with the second polarizer added in a second milled slot in the mounting bracket between the first polarizer and the source, and, d) with the first polarizer removed leaving only the second polarizer. At each wavelength of interest measurements are taken as a function of polarizer orientation as the polarizers are rotated about the optic axis. The first polarizer is typically sampled every 15° and the second every 30°. The second polarizer is tilted in its mount by about 10° to avoid polarizer-polarizer interreflections. All of these measurements are automated and require typically about an hour at each wavelength.

In the analysis of this data we write the first row Mueller matrix elements of the spectroradiometer responsivity as

$$R=R_{0}\left|\begin{array}{cccc} 1 & B & 0 & 0 \end{array}\right|$$ (1a)

where *R*_0_ is the ordinary responsivity neglecting polarization and we take the reference direction for polarization to be in whatever direction the radiometer polarizance lies, thus forcing the 45° component to vanish. The reference direction was determined to be horizontal by simply observing the effect of a hand-held polarizer of known polarization axis upon a lamp measurement. The polarization axis of the polarizer, in turn, was confirmed by viewing sunlight reflected at glancing incidence through the polarizer. Circular polarization is assumed to be negligible for the type of instrument and sources employed here. The quantity *B* represents the degree of linear polarization of the spectroradiometer. The Mueller matrix of a polarizer is taken to be of the form

$$P=\left|\begin{array}{cccc} s\; & dC\; & dS\; & 0\\ dC & eC^{2}+f & g\text{SC} & 0\\ dS & X & X & X\\ 0\; & X & X & X \end{array}\right|$$ (2a)

where C=cos 2(ϕ − ϕ_0_), S=sin 2(ϕ − ϕ_0_) and the parameters *s, d, e, f, g*, and ϕ_0_ are obtained by least-squares fitting of the measurements. The angle ϕ is the orientation angle of the polarizer read from a scale on the polarizer mount. The matrix elements marked X are not involved in these measurements. This form of the Mueller matrix was found, by trial and error, to fit our data well. The simpler model of an ideal dichroic polarizer in which *e=s* and *g=s−f* (where, in terms of the principle transmittances k_1_ and k_2_, we have *s* = [k_1_+k_2_]/2 and *d* = [k_1_ − k_2_]/2) is adequate for our visible and infrared polarizers but fails to fit the ultraviolet polarizer data. Finally, the Stokes vector of the unpolarized source is given by

$$L=L_{0}\left|\begin{array}{c} 1\\ 0\\ 0\\ 0 \end{array}\right|$$ (3a)

The various measurements then lead to equations of the following forms.

$$\text{No}\;\text{polarizer}:\;V=R\cdot L=R_{0}L_{0}$$ (4a)

$$\begin{array}{l} \text{Polarizer}\;\#1:\;V(\phi )=R\cdot P\cdot L\\ \;=R_{0}L_{0}(s+Bd\;C) \end{array}$$ (5a)

$$\begin{array}{c} \text{Both}\;\text{polarizers}:\;V(\phi ,\phi \prime )=R\cdot P\cdot P\prime \cdot L\\ =R_{0}L_{0}(ss\prime +Bs\prime d\;C+dd\prime CC\prime +Bd\prime fC\prime \\ +dd\prime \mathrm{SS}\prime +Bd\prime g\text{SC}S\prime +Bd\prime eC^{2}C\prime ) \end{array}$$ (6a)

$$\begin{array}{l} \text{Polarizer}\;\#2:\;V(\phi \prime )=R\cdot P\prime \cdot L\\ \;=R_{0}L_{0}(s\prime +Bd\prime C\prime ) \end{array}$$ (7a)

where C′=cos *2*(*ϕ*′ *− ϕ*_0_′), S′ = sin 2(*ϕ*′ *− ϕ*_0_′), and *V* represents the recorded radiometer signal output as a function, in general, of polarizer orientation angles. Altogether our measurements produce about 200 equations. The values of the ten unknowns *s, s*′, *d, d*′, *e,f, g, B, ϕ*_0_, and *ϕ*_0_′ are then obtained by a non-linear least squares solution. As far as polarization is concerned the parameters *s, d, e, f, g*, and *ϕ*_0_ completely characterize the first polarizer, at least within the accuracy of the model represented by the Mueller matrix of eq (10), and the parameter *B* characterizes the spectroradiometer. Since the factor *R*_0_*L*_0_ is common to all equations these measurements yield no information about it.

Using the characterized polarizer and radiometer we are now able to measure the polarization of an unknown source by recording spectroradiometer measurements obtained from the source: a) without a polarizer, and b) with the first polarizer again mounted on the bracket as when it was characterized. As before, the measurements are made at 15° intervals of the polarizer. We take the Stokes vector of the unknown source to be

$$L=L_{0}\left|\begin{array}{l} 1\\ p_{1}\\ p_{2}\\ 0 \end{array}\right|$$ (8a)

where, as always, we have neglected circular polarization. *p*_1_ and *p*_2_ are given by *p*_1_=*p*·cos2*τ* and *p*_2_*=p*·sin2*τ*, where *τ* is the direction of polarization measured from the reference direction of the radiometer and *p* is the degree of polarization,

p=(Lmax−Lmin)/(Lmax+Lmin). (9a)

In eq (17)*L*max and *L*min are the maximum and minimum readings of a polarization-indifferent radiometer when an ideal linear polarizer is rotated in the source beam. Then our measurements lead to a no-polarizer equation:

$$V=R_{0}L_{0}(1+Bp_{1})$$ (10a)

and to 24 equations:

$$V(\tau )=R_{0}L_{0}[(s+Bd\;C)+(d\;C+Be\;C^{2}+Bf)p_{1}+(d\;S+Bg\;\text{SC})p_{2}].$$ (11a)

Aside from the factor *R*_0_*L*_0_ which is common to all equations and cancels out, the only unknowns in these 25 equations are *p*_1_ and *p*_2_ which we can then obtain by a least-squares solution. These two parameters completely characterize the state of (linear) polarization of the source. Normally, only the second described experiment of 25 measurements need be performed to measure the polarization of a source since the polarizers and radiometer are relatively stable and, once characterized, will only infrequently need to be measured.

Equation (10a) gives the relationship between the spectral radiance of a source, *L*_0_, and the radiometer output signal, *V*, assuming linearity of response and only linear polarization. If we apply this equation both to the measurement of an unknown source and to the measurement of a spectral radiance standard used to calibrate the radiometer we obtain:

$$L_{0}=L_{0}^{s}[V/V^{s}][(1+Bp_{1}^{s})/(1+Bp_{1})]$$ (12a)

where $L_{0}^{s}$ is the known spectral radiance of the standard, *V/V^s^* is the measured signal ratio, *B* is the radiometer polarizance, and $p_{1}^{s}$ and *p*_1_ are the relative Stokes components measured as described above for the standard and for the unknown source. (For a blackbody standard, presumably, $p_{1}^{s}=0$.) This shows that the presence of polarization introduces the extra factor

$$\left(1+Bp_{1}^{s})/(1+Bp_{1}\right)$$

into the simple measurement relationship which is valid in the absence of source polarization or if the radiometer is indifferent to polarization.

The polarization measurement uncertainty by this process can be judged by the statistics of the least-squares fitting and by the measurement reproducibility when different polarizers are used. Both indications suggest typical uncertainties (one standard deviation) in the visible where the polarizers are very good and the noise level very low of ±0.0003 in the radiometer polarizance *B* and uncertainties of ±0.001 in the source relative Stokes components *p*_1_ and *p*_2_.
